# Both F-18 FDG-avidity and Malignant Shape of Cervical Lymph Nodes on PET/CT after Total Thyroidectomy Predict Resistance to High-dose I-131 Therapy in Patients with Papillary Thyroid Cancer

**DOI:** 10.7508/aojnmb.2013.01.003

**Published:** 2013

**Authors:** Byung Hyun Byun, Seong Young Kwon, Ari Chong, Jahae Kim, Su Woong Yoo, Jung-Joon Min, Ho-Chun Song, Henry Hee-Seung Bom

**Affiliations:** 1 Department of Nuclear Medicine, Chonnam National University Hwasun Hospital, Hwasun, Jeollanam-do, Republic of Korea; 2 Department of Nuclear Medicine, Chonnam National University Hospital, Gwangju, Republic of Korea; 3 President, AOFNMB

**Keywords:** F-18 FDG PET/CT, resistance to I-131 therapy, cervical lymph node, papillary thyroid cancer

## Abstract

**Objective:**

Resistance of metastatic lymph nodes (LNs) to high dose I-131 therapy is associated with high morbidity in patients with differentiated thyroid cancer. We evaluated the role of F-18 FDG PET/CT in the prediction of resistance to high dose I-131 therapy in patients with papillary thyroid cancer.

**Methods:**

The subjects were 307 patients who underwent total or near total thyroidectomy followed by high dose (5.55-6.66 GBq) I-131 therapy. We divided the patients into three subgroups by visual assessment of regional LNs: FDG-avid LNs with a malignant shape on CT (PET/CT-positive group), FDG-avid LNs with a benign shape on CT (PET/CT-intermediate group) and no FDG-avid lesion (PET/CT-negative group). We measured the maximum SUV (SUVmax) of FDG-avid LNs in each patient. The presence or absence of focal increased uptake of I-131 was evaluated by whole body scan (WBS), and was denoted as WBS-positive group or WBS-negative group, respectively. Resistance to therapy was defined as presence of thyroglobulin (Tg) in serum (Tg ≥1.0 ng/ml) 3-6 months after I-131 therapy. Univariate and multivariate analyses were performed to determine the relationship between resistance to I-131 therapy and various clinico-pathologic variables.

**Results:**

PET/CT-positive, intermediate, and negative groups included 20 (6.5%), 44 (14.3%) and 243 (79.2%) patients, respectively. The mean SUVmax was significantly higher in the PET/CT-positive group than that of the PET/CT-intermediate group (4.6 vs. 2.7, P <0.001). Univariate analysis revealed that the PET/CT-positive group (P <0.001), T2-4 stage (P <0.001), N1b stage (P = 0.001), lower dose (5.55 GBq) of I-131 (P <0.001), and the WBS-positive group (P = 0.029) were associated with resistance to therapy. In multivariate analysis, the PET/CT-positive group, lower dose of I-131, N1b stage, and T2-4 stage remained significant with odds ratios of 10.07 (P <0.001), 3.82 (P <0.001), 3.58 (P = 0.001), and 2.53 (P = 0.009), respectively.

**Conclusion:**

FDG-avidity and malignant shape of cervical LNs on pre-therapy FDG PET/CT were a strong risk factors predicting resistance to high dose I-131 therapy. A lower dose of administered I-131 (5.55 GBq) and more extensive tumors (T2-4 and N1b) were also associated with resistance to high dose I-131 therapy.

## Introduction

Differentiated thyroid cancer (DTC) usually has a good prognosis (1). Among DTCs, papillary thyroid cancer (PTC) has been reported to metastasize to lymph nodes (LNs) more often than follicular thyroid cancers (2, 3), causing adverse effects on prognosis in patients with DTC (4).

Radioactive iodine (I-131) therapy following total or subtotal thyroidectomy has an ability to destroy loco-regional, and distant metastatic lesions as well as remnant thyroid tissue in patients with DTC (5-7). FDG PET/CT has been used for the detection of loco-regional or distant metastases of DTC (8, 9). Although I-131 ablation therapy is highly effective in the treatment of LN metastases from DTC (10), FDG-avid metastatic thyroid cancer concentrates little I-131, and hence is refractory to I-131 ablation therapy (11, 12).

It is common to find inflammatory or reactive cervical LNs in patients with DTC. FDG-avidity in those lesions can influence data analysis. We hypothesized that FDG-avid LNs with a malignant shape on CT are resistant to I-131 therapy while those with a benign shape on CT are not resistant. Along with visual assessment of FDG-avidity of LNs, clinico-pathological variables and the dose of administered I-131 were also used to predict resistance to high dose I-131 therapy.

## Methods

### Patients

We retrospectively reviewed the clinical records and images of patients with PTC who were treated at our institution between January 2008 and March 2011. Patients who met all of the following criteria were included in this study: Patients with PTC who underwent total or near total thyroidectomy, and central compartment LN dissection with or without laterocervical compartment LN dissection; the first I-131 therapy (5.55 GBq or 6.66 GBq) at least 2 months after surgery; N1a or N1b on postsurgical specimen; No history of other primary malignancies at the time of I-131 therapy; No evidence of distant metastasis (M0); Serum antithyroglobulin antibody (TgAb) ≤60 UI/ml (to preclude the possible alteration of thyroglobulin level) (13); FDG PET/CT was performed within one week before I-131 therapy. To focus on the association between FDG PET/CT findings of regional LN metastasis, and success rate of I-131 therapy, we included only PTC patients with N1 stage without distant metastasison FDG PET/CT, post-therapeutic I-131 whole body scan (WBS) or other studies. The TNM stages were based on the seventh edition of the AJCC Cancer Staging Manual (14).

All patients were prepared for I-131 therapy by withholding levothyroxine (T4) for 4 weeks, replacing with triiodothyronine (T3) for the first 2 weeks of the study period, followed by a low iodine diet for the next 2 weeks. They were admitted for 3-4 days, and underwent assays for thyroglobulin (Tg), TgAb, and thyroid stimulating hormone (TSH) on the first day of admission. The patients were treated with a dose of 5.55 GBq I-131 between January 2008 and March 2010, and the dose was raised to 6.66 GBq between April 2010 and March 2011. Serum Tg, TgAb, and TSH were also measured 3-9 months after I-131 therapy with TSH suppression (TSH <1 µIU/mL). ‘Tg-negative’ was defined as Tg <1.0 ng/mL with TgAb ≤60 UI/ml, and ‘Tg-positive’ was defined as Tg ≥1.0 ng/mL. Tg-positive result after 3-9 months of high dose I-131 therapy was regarded as resistance to I-131 therapy. This retrospective study was approved by our institutional review board.

### Imaging procedures

FDG PET/CT scans were performed with a Discovery STE PET/CT system (GE Medical Systems, Milwaukee, WI, USA) following the standard protocol used at the institution. Briefly, all patients fasted for at least 6 h before the intravenous administration of 7.4 MBq/kg body weight of FDG. Blood glucose level did not exceed 7.2 mmol/L in any patient. No iodinated contrast was administered intravenously. CT images were acquired from the skull base to the upper thigh using the following parameters: 120 kV peak voltage, 10–130 mAs automated tube current, 0.8 s rotation time, 0.5 m field of view, 40–50 s scan, and 3.75 mm slice thickness. Immediately after the CT acquisition, PET data was acquired in the same anatomic locations, with a 15.7 cm axial field of view in the 3D mode at 150 s/bed position. The CT data was used for attenuation correction, and the images were reconstructed using a conventional iterative algorithm (ordered-subsets expectation-maximization). A workstation (AW Volume Share™, GE Healthcare) providing multiplanar reformatted images was used for image display and analysis.

Post-therapeutic I-131 WBS was obtained on the 3rd (WBS-3d), and 7th day (WBS-7d) after I-131 therapy using two variable-angle dual-head gamma cameras (Millennium VG and Infinia Hawkeye 4, GE Medical Systems, Milwaukee, WI, USA). The instruments had parallel-hole high-energy collimators, with a matrix size of 512 × 512 and a 364-keV photopeak with 10% windows.

### Data analysis

On axial PET images, regional LNs with focally increased FDG accumulation compared to adjacent normal tissue were selected as the region of interest (ROI), and the maximum SUV (SUVmax) were generated over the ROI. CT images at the same level were evaluated, and LN metastases were suggested when they satisfied these CT criteria: LNs of spherical shape, and minimal axial diameter >10 mm, or LNs with scattered calcification and/or cystic change (15).

**Figure 1:**
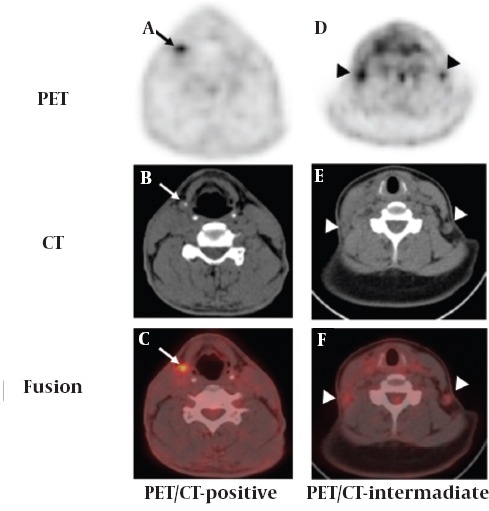
Representative cases of F-18 fluorodeoxyglucose (FDG) positron emission tomography/computed tomography (PET/CT). A PET/CT-positive case (A-C) involves a 48-year-old male showing a focal FDG uptake in the right cervical lymph nodes with the maximum SUV of 5.6 on PET (black arrow in A) and a malignant shaped LN, i.e. spherical LNs with eccentric calcification, suggesting metastasis on CT (white arrow in B). A PET/CT-intermediate case (D-F) involves a 47-year-old female showing a focal FDG uptake in both cervical LNs with the maximum SUV of 3.4 (left) and 3.8 (right) on PET (black arrowhead in D) and a benign shaped LN, i.e. an intact fatty hilum concaving into the central portion of LNs on CT (white arrowhead in E)

We divided the patients into three subgroups on the basis of FDG PET/CT findings: 1- PET/CT-positive group: regional LNs with increased FDG accumulation satisfying the CT criteria suggestive of LN metastasis (Fig. 1A-C), 2-PET/CT-intermediate group: regional LNs with increased FDG accumulation and nonsatisfaction of any of the CT criteria (Fig. 1D-F), and 3- PET/CT-negative group: no FDG-avid lesion on any of the CT findings.

**Figure 2:**
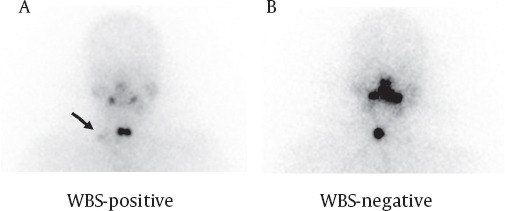
Representative cases of I-131 whole body scan (WBS). (A) A WBS-positive case involves a 39-year-old female showing a focal I-131 accumulation in the right lateral neck (arrow) as well as central neck uptakes. (B) A WBS-negative case involves a 37-year-old female showing a hot spot in the central neck but no I-131 accumulation in the lateral neck.

We also divided the patients into two subgroups on the basis of WBS findings: 1- WBS-positive group: focal I-131 accumulation in the regional neck outside the central neck on WBS-3d or WBS-7d (Fig. 2A), 2- WBS-negative group: no significant I-131 accumulation in the regional LNs (Fig. 2B). All FDG PET/CT and WBS images were visually interpreted by consensus of two experienced nuclear physicians.

### Statistical analysis

We compared the SUVmax of the PET/CT-positive group and intermediate group by unpaired t-test. Univariate analyses were performed to determine the association between Tg-positive rate after I-131 therapy, and other variables, such as age, gender, dose of administered I-131 (5.55 GBq and 6.66 GBq), and dichotomized variables (T and N stages, PET/CT and WBS subgroups). Independent variables with P <0.25 from the univariate analysis were included in the multivariate logistic regression analysis, and the results were considered statistically different if a P value was less than 0.05, and the 95% confidence interval of the odds ratio did not include in the multivariate analysis.

We compared the Tg-positive rate after I-131 therapy according to the PET/CT and WBS subgroups by Chi-square test or Fisher’s exact test. Additionally, we calculated the Tg-positive rate after I-131 therapy according to the number of risk factors determined from multivariate analysis. The statistical software package for social science (SPSS) version 19.0 for windows (SPSS Inc., an IBM Company) was used for all analyses.

## Results

### Patients Characteristics

Table 1 shows the characteristics of enrolled patients with PTC. Among 307 patients, 180 patients (58.6%) were over the age of 45, and 230 patients (74.9%) were women. More than a half of the patients had T1 (63.5%), and N1a (81.4%) disease on their postsurgical specimen. About 20% of the patients showed FDG-avid regional LNs, and approximately one-third of them (n=20) were categorized into the PET/CT-positive group. All patients had an elevated TSH >30 µIU/mL (median: 85.7 µIU/mL, range: 34.6-99.9 µIU/mL) on the day of I-131 therapy. A dose of 5.55 GBq I-131 was administered to 127 patients, and 6.66 GBq was administered to 180 patients. WBS revealed regional LN metastases in 17 patients (5.5%). The Tg-positive rate was 58.6% before I-131 therapy, and 16.3% after I-131 therapy. In addition, there was no case with TgAb >60 UI/ml among patients with Tg less than 1.0 ng/mL on follow-up study. The median serum Tg level before I-131 therapy was 1 ng/ml (range: 0-85ng/ml) in Tg-negative patients (n = 257), and 19 ng/ml (range:0-500ng/ml) in Tg-positive patients (n = 50).

**Table 1:**
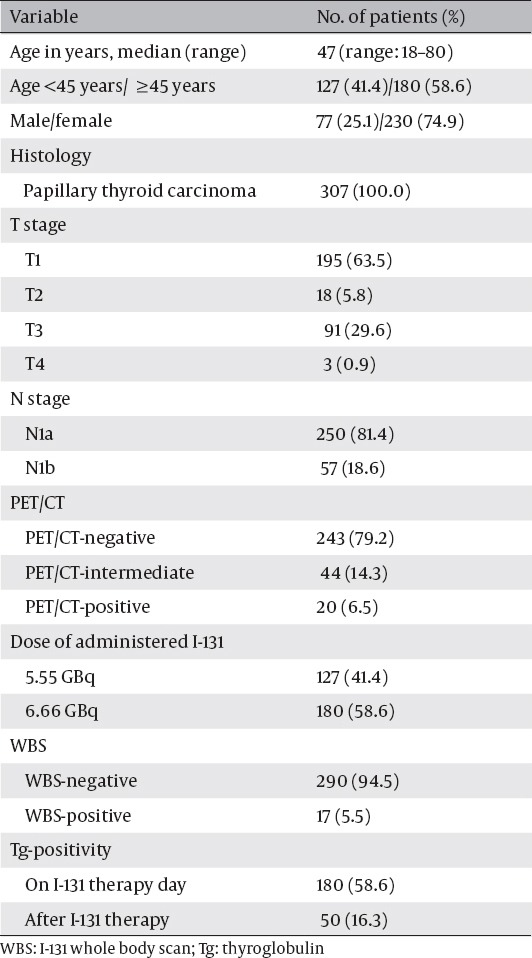
Characteristics of 307 Patients with Papillary Thyroid Cancer Enrolled in This Study

**Figure 3:**
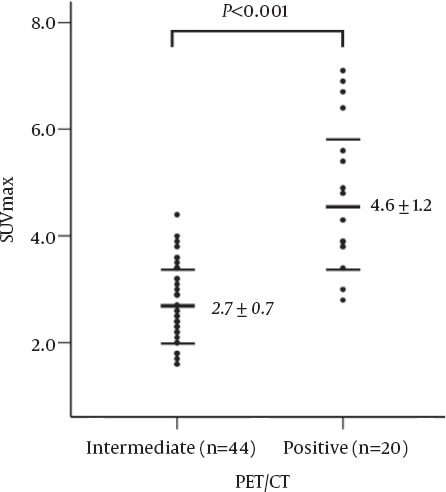
The maximum SUV (SUVmax) in the regional lymph nodes from the F-18 FDG PET/CT (PET/CT)-intermediate and PET/CT-positive groups. Although the mean value of SUVmax is significantly higher in the PET/CT-positive group than that in the PET/CT-intermediate group (P <0.001, unpaired t-test), a significant overlap can be noted.

Although the mean value of SUVmax of the PET/CT-positive group was significantly higher than that of the PET/CT-intermediate group (mean 4.6 vs. 2.7, P <0.001), the significant portion of SUVmax appeared to overlap with each other (Fig. 3).

Comparison of Independent Variables With the Tg-Positive Rate After I-131 Therapy

**Figure 4:**
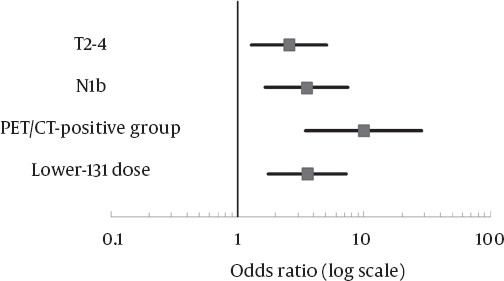
Odds ratio and 95% confidence interval (CI) of risk factors. PET/CT-positive group: FDG-avid lymph nodes with a malignant shape on FDG PET/CT; Lower I-131 dose: 5.55 GBq; T2-4: T stage 2 to 4; N1b: nodal stage 1b

Table 2 shows univariate and multivariate analyses of independent variables associated with the Tg-positive rate after I-131 therapy. Univariate analysis showed that T2-4, N1b, PET/CT-positive group, WBS-positive group, and lower dose of administered I-131 (5.55 GBq) were associated with the Tg-positive rate after I-131 therapy. T2-4, N1b, lower dose of administered I-131 (5.55 GBq), and PET/CT-positive group remained significant in multivariate analysis with odds ratios of 2.53 (P = 0.009), 3.58 (P = 0.001), 3.82 (P <0.001), and 10.07 (P <0.001), respectively (Fig. 4).

**Table 2:**
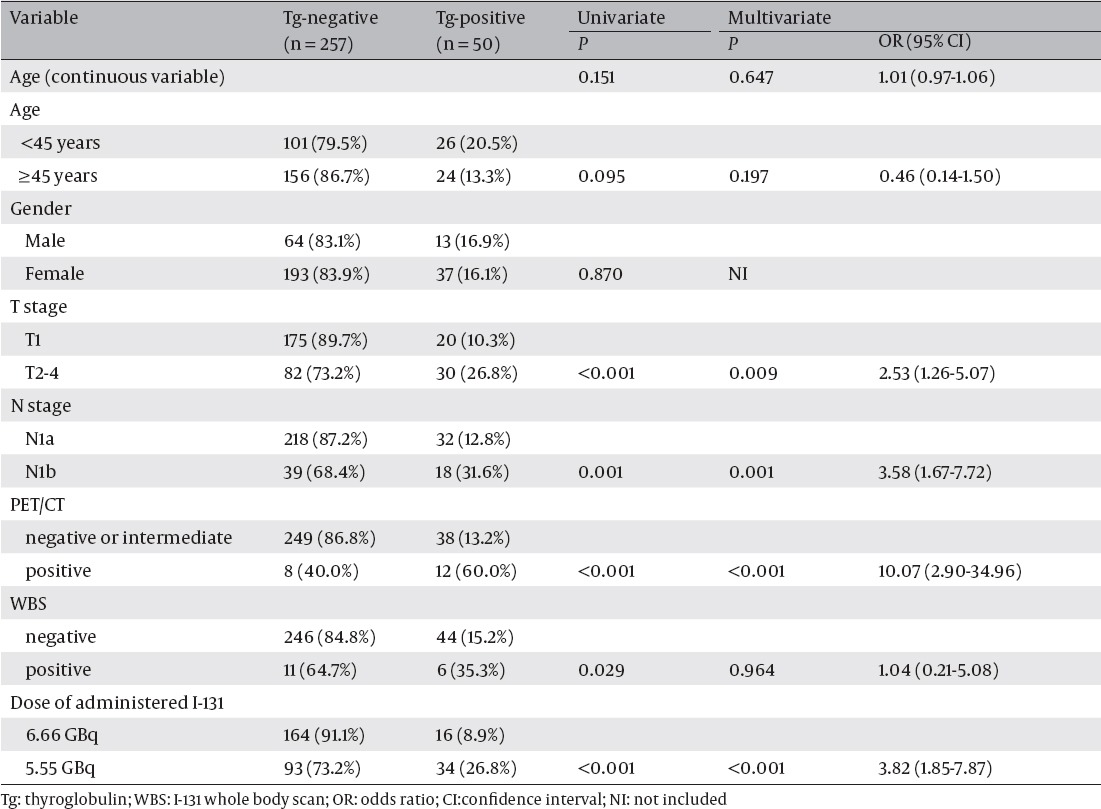
Univariate and Multivariate Analyses of Clinical Variables for Prediction of Thyroglobulin-Positivity After I-131 Therapy

Although the increase of I-131 dose from 5.55 GBq to 6.66 GBq in the PET/CT-positive group increased the Tg-negative rate from 27.3% (3 of 11) to 55.6% (5 of 9), but it was not statistically significant (P = 0.362).

Table 3 shows the Tg-positive rate after I-131 therapy according to the PET/CT and WBS subgroups. Tg-positive rate after I-131 therapy in the WBS-positive group (6 of 17, 35.3%) was higher than that in the WBS-negative group (44 of 290, 15.2%, P = 0.029). There were no statistically significant differences between the WBS-positive and negative groups when we subdivided the subjects according to the PET/CT findings. Especially, eight (40.0%) of 20 patients in PET/CT-positive group showed WBS positivity, compared to other PET/CT subgroups.

**Table 3:**
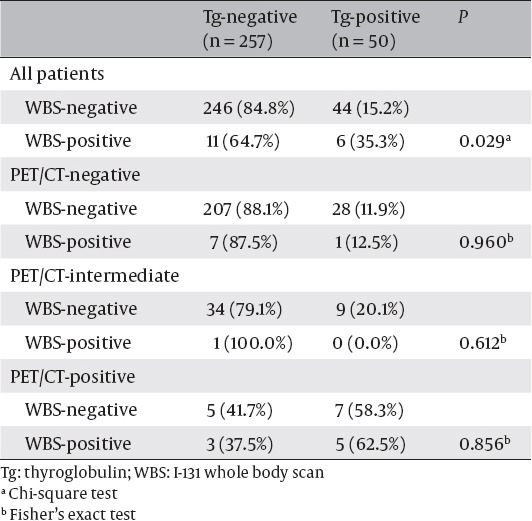
Thyroglobulin-Positivity After I-131 Therapy According to the FDG PET/CT and WBS Subgroups

Table 4 shows the Tg-positive rate after I-131 therapy according to the number of risk factors. On the basis of univariate analysis, three independent risk factors for resistance to therapy (T2-4, N1b, and PET/CT-positive group) were chosen. Only 8 of 156 patients (5.1%) without any risk factors showed Tg-positivity after I-131 therapy, while all three patients (100.0%) having all of the three risk factors showed Tg-positivity after I-131 therapy. Patients with one or two risk factors showed intermediate rates of Tg-positivity.

**Table 4:**
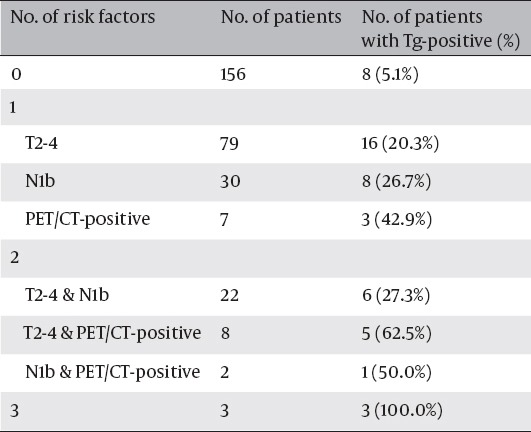
The Frequency of Thyroglobulin-Positivity After I-131 Therapy According to the Numbers of Risk Factors for Resistance to Therapy (T2-4 Stage, N1b Stage, and PET/CT-Positive Group)

## Discussion

In this retrospective study, we observed that patients with FDG-avid regional LNs with CT findings of metastasis were resistant to high dose I-131 therapy, while patients with FDG-avid regional LNs without CT findings of metastasis showed similar Tg-positive rates to LNs with no FDG uptake. Therefore, FDG PET/CT before high dose I-131 therapy has a clinical role if both PET and CT images are carefully evaluated. Besides the PET/CT findings, a lower dose of administered I-131 (5.55 GBq compared to 6.66 GBq), and more extensive tumors (i.e., T2-4 and N1b stages compared to T1 and N1a) were also associated with resistance to I-131 therapy.

It has been reported that DTC without remaining functional differentiation for hormone synthesis, and iodine uptake have high glucose metabolism (16, 17), and high-dose I-131 therapy has little or no effect on the viability of metastatic FDG-avid thyroid cancer lesions (12, 18). Although the accumulation of FDG reflects the regional tumor metabolism, it is not very tumor-specific, and many inflammatory lesions with increased glucose metabolism may cause false-positive results in FDG PET/CT (19). FDG-avid LNs of the PET/CT-intermediate group might have resulted from inflammatory lesions, and these LNs were not expected to have influenced the therapeutic effect of I-131. Only one (2.3%) of 44 PET/CT-intermediate LNs showed WBS positivity, and it indicates that most PET/CT-intermediate LNs were inflammatory rather than malignant LNs without iodine uptake (Table 3).

There are several reports in the literature indicating that tumor stage is a significant predictor of successful therapy in patients with DTC (20, 21). It is reasonable to assume that patients with advanced stage have a larger number of malignant tumor cells than those with limited stage. Thus, more frequent rate of Tg-positivity in patients with T2-4 or N1b compared to those with T1 or N1a in this study is consistent with previous reports. These two variables remained significant in multivariate analysis, indicating that T and N stages were independent predictors of resistance to I-131 therapy. However, T and N stages are not enough to predict the therapeutic outcome precisely because these parameters could not reflect metabolic characteristics of cancer. Therefore, the prediction of therapeutic effectiveness by evaluating FDG avidity in LN could have important implications for the risk stratification of PTC patients and decision of next treatment plan. For example, alternative approaches such as redifferentiation therapy using retinoic acid in combination with I-131 (22, 23), surgical removal of metastatic LNs, radiotherapy, or tyrosine kinase inhibitor (24) can be considered as treatment options in PET/CT-positive group (25).

The WBS-positive group showed a more frequent rate of Tg-positivity compared to the WBS-negative group in univariate analysis but not significant in multivariate analysis. There could be several factors to explain these results. First of all, nine of 17 WBS-positive group showed FDG avidity simultaneously, and eight showed PET/CT-positive results (Table 3). I-131 therapy was less successful in the PET/CT-positive group, and more successful in the PET/CT-negative group regardless of the WBS findings. These results indicate that FDG-avid metastasis rather than iodine-avid metastasis is refractory to I-131 therapy, which are in line with previous studies (11, 12, 26).

The optimal therapeutic dose of I-131 for DTC with locoregional or distant metastasis remains uncertain and controversial (27-29). One of the most frequently used criteria of empiric fixed dose (5.5-7.4 GBq) was proposed by Beierwaltes et al. (29). In the present study involving only PTC patients with N1M0, we observed a three times lower rate of successful therapy (9% vs. 27%) in the lower dose (5.55 GBq) group compared to the higher dose (6.66 GBq) group. The dose was determined by the date of treatment. A lower dose was administered in the first half, and a higher dose in the latter half of the study period, providing minimum randomness of the study. According to the observation of this study, authors now choose 6.66 GBq of I-131 for PTC patients with stage N1M0. A further increase of the I-131 dose can be an option in the PET/CT-positive group; although, we could not obtain a statistically significant result in this study. These results have some points in conflict with a recent clinical trial showing no significant difference of response rate between 1.11 GBq and 3.70 GBq of I-131, even if different population and methods of our study were considered (30). Prospective clinical trials with larger number of patients could help to decide optimal dose of I-131 therapy in each country.

There are several limitations in this retrospective study. First of all, Tg after I-131 therapy was measured without TSH stimulation, and about 20% of patients with Tg<1 ng/mL during TSH suppression were reported that Tg was elevated more than 2 ng/mL during TSH stimulation (31). Considering these previous studies, the success rate of high dose I-131 therapy in our study might have been overestimated. On the other hand, the success rate of high dose I-131 therapy might have been underestimated because there could be false-positive results of Tg without residual thyroid tissue or metastatic LNs. Moreover, follow-up period was not enough in our study, and Tg level before I-131 therapy was not considered. Therefore, ‘Tg ≥1.0 ng/mL’ might have been in the process of falling, especially in patients with high Tg level before I-131 therapy. In addition, Tg alone may not be sufficient to evaluate therapeutic response. Other points were several cases ambiguous to characterize LNs on CT, because CT data was used for attenuation correction instead of diagnostic purpose and, moreover, CT images were acquired with a variable range of dose (10-130 mAs). These CT protocol could give suboptimal CT information for the definition of LN morphology.

## Conclusion

We investigated the predictive role of FDG PET/CT findings, i.e. FDG avidity and malignant shape of LNs, for resistance to I-131 therapy in PTC patients with N1M0 stage. Our data indicated that FDG-avid LNs with CT findings of metastasis in PTC patients were a strong predictor for resistance to I-131 therapy independent of clinico-pathological variables. Theserisk factors can be useful for the risk stratification of PTC patients undergoing I-131 therapy, and decision of next therapeutic management.
